# Triclosan causes spontaneous abortion accompanied by decline of estrogen sulfotransferase activity in humans and mice

**DOI:** 10.1038/srep18252

**Published:** 2015-12-15

**Authors:** Xiaoli Wang, Xiaojiao Chen, Xuejiao Feng, Fei Chang, Minjian Chen, Yankai Xia, Ling Chen

**Affiliations:** 1 State Key Lab of Reproductive Medicine, Nanjing Medical University, Nanjing 210029, China; 2 Department of Physiology, Nanjing Medical University, Nanjing 210029, China; 3 Nanjing Maternity and Child Health Hospital, Nanjing Medical University, Nanjing 210029, China; 4 Key Laboratory of Modern Toxicology of Ministry of Education, School of Public Health, Nanjing Medical University, Nanjing 210029, China

## Abstract

Triclosan (TCS), an antibacterial agent, is identified in serum and urine of humans. Here, we show that the level of urinary TCS in 28.3% patients who had spontaneous abortion in mid-gestation were increased by 11.3-fold (high-TCS) compared with normal pregnancies. Oral administration of TCS (10 mg/kg/day) in mice (TCS mice) caused an equivalent urinary TCS level as those in the high-TCS abortion patients. The TCS-exposure from gestation day (GD) 5.5 caused dose-dependently fetal death during GD12.5–16.5 with decline of live fetal weight. GD15.5 TCS mice appeared placental thrombus and tissue necrosis with enhancement of platelet aggregation. The levels of placenta and plasma estrogen sulfotransferase (EST) mRNA and protein in TCS mice or high-TCS abortion patients were not altered, but their EST activities were significantly reduced compared to controls. Although the levels of serum estrogen (E2) in TCS mice and high-TCS abortion patients had no difference from controls, their ratio of sulfo-conjugated E2 and unconjugated E2 was reduced. The estrogen receptor antagonist ICI-182,780 prevented the enhanced platelet aggregation and placental thrombosis and attenuated the fetal death in TCS mice. The findings indicate that TCS-exposure might cause spontaneous abortion probably through inhibition of EST activity to produce placental thrombosis.

Spontaneous abortion is the most common complication of human pregnancy and 10–15% of clinical pregnancies end in it^1^. Recently, a growing body of evidence has supported an association between exposure to environmental endocrine disrupters and spontaneous abortion^2^. Triclosan (TCS), an antibacterial agent, is widely used in soaps, toothpastes, first-aid products, fabrics and plastic goods^3^,^4^. This compound has been identified in the mother’s milk, the plasma of people in Sweden and Australia^5^,^6^ and the urine of people in the United States^7^.

There are several biological activities of TCS that are unrelated to its antibacterial action. For example, TCS reduces the level of thyroid hormone in weanling rats^8^,^9^, and exhibits estrogenic and androgenic activities in breast cancer cells^10^. Triclosan is known to inhibit sulfonation of phenolic xenobiotics in human liver cytosol and is structurally related to inhibitors of estrogen sulfotransferase (EST)^11^. James *et al.* has reported that TCS can inhibit the activity of sheep placental cytosolic EST to reduce the levels of both estradiol and estrone sulfonation^12^. EST, encoded by *SULT1E1,* is expressed in human, bovine and murine placentas during mid- to late gestation to catalyze the sulfoconjugation of estrogen (E2) at the 3-hydroxyl position^13^,^14^,^15^. The discrete localization of EST at the interface of the fetal-maternal blood exchange suggests that EST may play a critical role in modulating E2 activity in both fetal and maternal plasma^16^. Either genetic or chemical inactivation of placental EST may cause pregnancy failure or intrauterine growth retardation in humans and other mammals^17^. Exposure to TCS at an average dose of 3.2 mg/kg has been reported to decrease the survival of postnatal mice^18^. However, the effects of TCS-exposure during mid- to late gestation on the maintenance of pregnancy and fetal development have not yet been reported.

To determine the influence of TCS on the process of pregnancy, an epidemiological investigation was primarily designed to examine the level of urinary TCS in normal pregnancy and spontaneous abortion patients in mid-gestation. According to the level of urinary TCS in spontaneous abortion patients, we prepared the model of pregnant mouse exposed to TCS, in which the fetal survival and development were examined during mid- to late gestation. In addition, placental structure, the levels of reproductive hormones and thyroid hormones, the expression and activity of EST were further examined in spontaneous abortion patients and TCS-exposed pregnant mice. Our results indicated that the exposure of TCS in humans and mice might cause spontaneous abortion in mid-gestation probably through the inhibition of EST activity leading to placental thrombosis and degeneration.

## Results

### Spontaneous abortion patients in mid-gestation with a high level of urinary TCS

Measuring TCS and its metabolites in urine represents an important biomonitoring tool for exposure assessment^19^. The baseline excretion of TCS in urine was published by Sandborgh-Englund *et al.*^20^. In the present study, all 452 eligible females provided urine for analysis. There were 113 spontaneous abortion patients and 339 normal gestations in mid-gestation weeks 14–24, with a mean age of 28.30 ± 4.24 years and 27.55 ± 3.90 years, respectively. The smoking status in abortion patients had no difference from control group (*P* = 0.492). Meanwhile, there was a difference in the body mass index (BMI) between abortion patients (21.89 ± 2.60 kg/m^2^) and controls (23.49 ± 3.62 kg/m^2^; *P* = 0.038). In abortion patients, the detectable rate of urinary TCS (57.52%; Table 1) was 1.76-fold higher as compared to control group (32.74%). In addition, the mean concentration of urinary TCS in abortion patients (2.50 ng/ml) was increased approximately 2.53 folds compared to control group (0.99 ng/ml). The crude and adjusted odds ratios (ORs) for the association between the TCS exposure level and spontaneous abortion were shown in the Table 2. A dose-response relationship was observed for TCS exposure [adjusted ORs for increasing exposure levels = 1.00, 2.00 (1.08–3.70), 2.36 (1.29–4.34); *P* value of test for trend = 0.003]. Particularly, the level of urinary TCS in 28.3% abortion patients (11.21 ng/ml) was increased approximately 11.3-fold compared to mean of controls (0.99 ng/ml).

### TCS-exposure causes spontaneous abortion in mid-gestation

**Figure 1:**
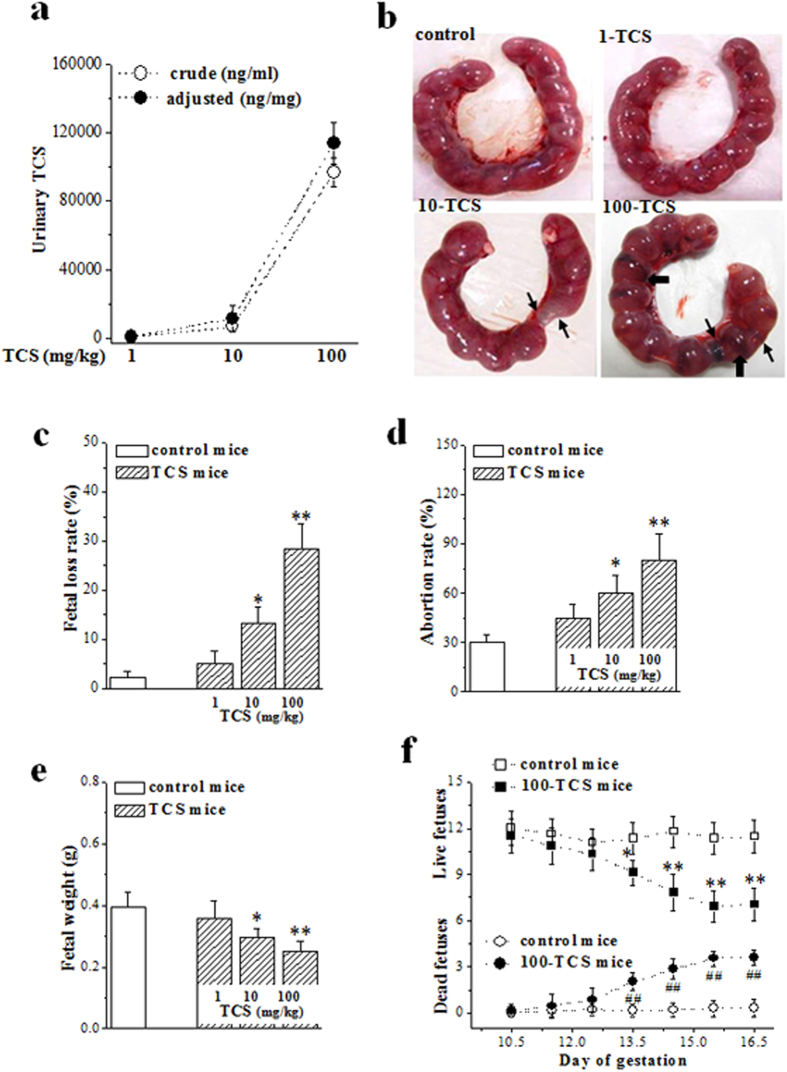
TCS-exposure causes spontaneous abortion in mid-gestation. (**a**) Levels of TCS in urine of GD10.5 mice exposed to various doses of TCS. Adjusted concentration: adjusted for the creatinine level. (**b**) Representative picture of fetuses in GD15.5 control mice, mice exposed to 1 mg/kg TCS (1-TCS), 10 mg/kg TCS (10-TCS) and 100 mg/kg TCS (100-TCS). Thick arrows: fetal death; thin arrows: reabsorbed fetuses. (**c**) Ratio of lost fetuses against total number of fetuses in GD15.5 TCS mice. *P < 0.05 and **P < 0.01 *vs*. control mice (one way ANOVA). (**d**) Ratio of spontaneous abortion in GD15.5 control mice and TCS mice. *P < 0.05 and **P < 0.01 *vs*. control mice (one way ANOVA). (**e**) Body weight (g) of live fetuses in GD15.5 control mice and TCS mice. *P < 0.05 and **P < 0.01 *vs*. control mice (one way ANOVA). (**f**) Average number of live (squares) and dead (circles) fetuses in control mice (open symbols) and TCS mice (filled symbols) at GD10.5-16.5. *P < 0.05 and **P < 0.01 (live fetuses) *vs*. control mice; ^##^*P* < 0.01 (dead fetuses) *vs*. control mice (two way ANOVA).

To examine the influence of TCS on the maintenance of pregnancy and fetal development, 3-month-old mice were treated with TCS daily by gavage at doses of 1, 10 and 100 mg/kg/day (termed 1-TCS mice, 10-TCS mice and 100-TCS mice, respectively) from gestation day (GD) 5.5. The level of urinary TCS was measured at GD10.5 and GD15.5, respectively. As shown in Fig. 1a, the level of urinary TCS in GD10.5 10-TCS mice was equivalent to that in abortion patients with a high exposure to TCS (high-TCS). The correlation analysis showed a positive linear correlation between the urinary TCS levels and the TCS-exposure doses (R^2^ = 1, n = 20). However, the level of urinary TCS showed no significant difference between GD10.5 and GD15.5 10-TCS mice (data not shown).

The TCS-exposure caused a dose-dependent increase in the fetal death (Fig. 1b). In comparison with GD15.5 control mice, the number of live fetuses was reduced by approximately 30% in 100-TCS mice (*P* < 0.001, n = 20; Fig. 1c) and 15% in 10-TCS mice (*P* = 0.030, n = 20), respectively. The incidence of spontaneous abortion in 100-TCS mice and 10-TCS mice reached 80% (*P* < 0.001, n = 20; Fig. 1d) and 60% (*P* = 0.015, n = 20), respectively. In addition, the body weight of live fetuses was significantly reduced in 100-TCS mice (*P* < 0.001, n = 20; Fig. 1e) and 10-TCS mice (*P* = 0.018, n = 20) compared with controls.

To determine the time of TCS-induced spontaneous abortion, we further examined the numbers of live and dead fetuses during GD10.5-16.5. As shown in Fig. 1f, the reduction of live fetuses was primarily observed at GD13.5 (*P* = 0.015, n = 10) and was gradually aggravated during GD14.5 (*P* < 0.001, n = 10) and GD16.5 (*P* < 0.001, n = 10). Consistently, the number of dead or reabsorbed fetuses showed a progressive increase from GD13.5 (*P* = 0.004, n = 10) to GD16.5 (*P* < 0.001, n = 10).

### TCS-exposure enhances placental thrombosis

**Figure 2:**
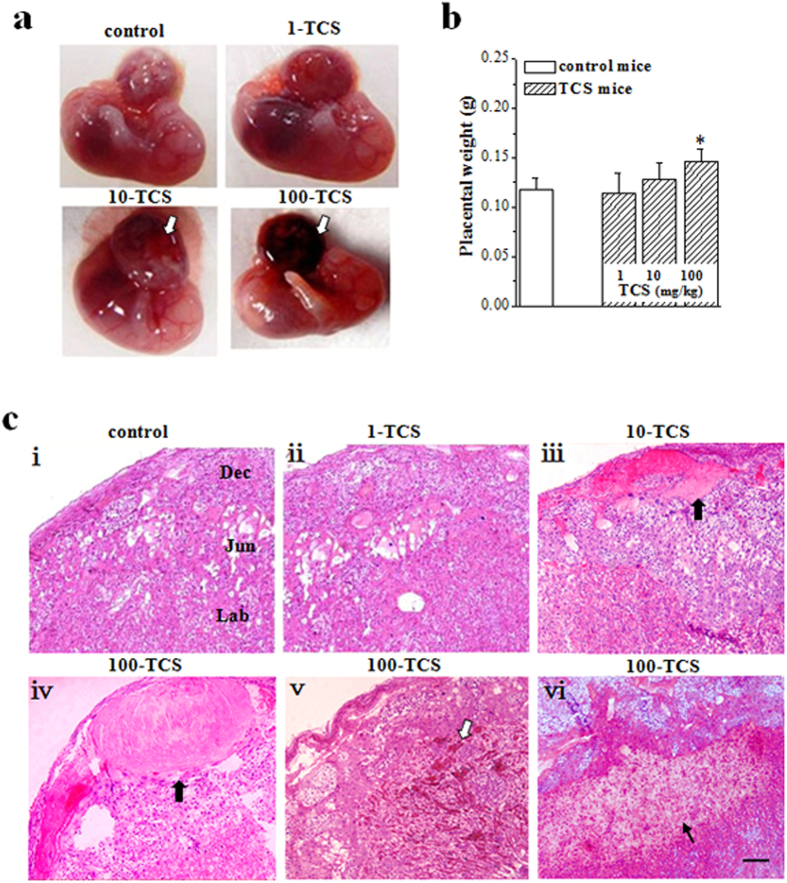
TCS-exposure causes placental thrombosis and hemorrhage. (**a**) Representative picture of placentas in GD15.5 control mice and TCS mice. (**b**) Placenta weight (g) in GD15.5 control mice and TCS mice. *P < 0.05 *vs*. control mice (one way ANOVA). (**c**) Representative picture of placental histological observation by hematoxylin and eosin staining in GD15.5 control mice and TCS mice. Lab: labyrinthine zone; Jun: junctional zone; Dec: deciduas. Thick arrows: thrombi; thin arrow: tissue necrosis; hollow arrow: hemorrhage area. Scale bars = 100 μm.

Notably, the placentas were dark in GD15.5 10-TCS mice and 100-TCS mice, but not in 1-TCS mice (Fig. 2a). The placental weight in GD15.5 100-TCS mice had a slight increase compared to control mice (*P* = 0.015, n = 20; Fig. 2b). The histological observation showed the placental thrombus (Fig. 2c–iii,iv) and hemorrhage (Fig. 2c–v) with tissue necrosis (Fig. 2c–vi) and atrophy of junctional zone in approximately 50% of 10-TCS mice and nearly all 100-TCS mice.

### TCS-exposure alters the levels of reproductive hormones and thyroid hormones

The levels of serum estrogen (E2), progesterone (P4) and β-human chorionic gonadotropin (β-HCG) were investigated in 100-TCS mice and abortion patients. The levels of E2 (*P* = 0.358, n = 20; Table 3), P4 (*P* = 0.196, n = 20) and β-HCG (*P* = 0.419, n = 20) in GD12.5 TCS mice did not differ significantly from control mice. The levels of P4 (*P* < 0.001, n = 20) and β-HCG (*P* < 0.001, n = 20) were significantly reduced in GD15.5 100-TCS mice compared to controls, while E2 level had no significant difference between the both groups (*P* = 0.105, n = 20). However, the levels of sulfo-conjugated (S-E2) were reduced in TCS mice (GD12.5: *P* = 0.008, n = 20; GD15.5: *P* = 0.005, n = 20), while the levels of unconjugated E2 (free E2) were observably increased in TCS mice (GD12.5: *P* = 0.027, n = 20; GD15.5: *P* = 0.021, n = 20) compared to controls. Thus, the ratio of S-E2 and free E2 in TCS mice (GD12.5: 1.47 ± 0.83; GD15.5: 1.51 ± 0.57) was lower than that in control mice (GD12.5: 5.53 ± 1.32, *P* = 0.004; GD15.5: 5.12 ± 1.07, *P* < 0.001). In addition, the levels of serum thyroxine (T4) and triiodothyronine (T3) in TCS mice (GD12.5: *P* = 0.048, n = 20; GD15.5: *P* = 0.026, n = 20) were lower than those in control mice.

In the low-TCS and high-TCS abortion patients, the levels of P4 (low-TCS: *P* < 0.001, n = 48; high-TCS: *P* < 0.001, n = 32) and β-HCG (low-TCS: *P* < 0.001, n = 48; high-TCS: *P* < 0.001, n = 32) were clearly lower than those in normal gestation (control group, n = 339). The level of E2 in low-TCS abortion patients was lower than control (*P* = 0.012, n = 48), whereas in high-TCS abortion patients had no difference from control group (*P* = 0.074, n = 32).

### TCS-exposure suppresses EST activity to increase activated E2

**Figure 3:**
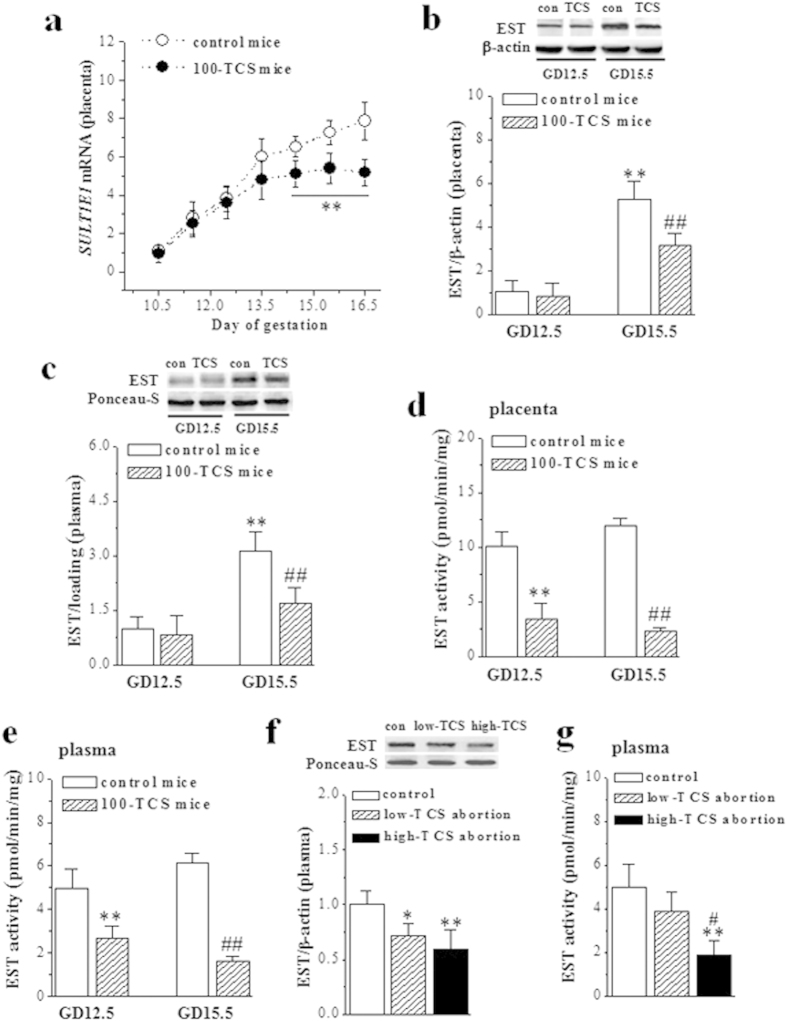
TCS-exposure suppresses the activity of EST. (**a**) Level of placental *SULT1E1* mRNA in GD10.5-16.5 control mice and 100-TCS mice. **P < 0.01 *vs*. control mice (two way ANOVA). (**b,c**) Representative immunoblotting and corresponding densitometric analysis showing the levels of placental and plasma EST protein in GD12.5 and GD15.5 control mice and 100-TCS mice. The blots in panel band were performed on the same blot membranes and shown as cropped images. **P < 0.01 *vs*. GD12.5 control mice. ^##^*P* < 0.01 *vs*. GD15.5 control mice (two way ANOVA). (**d,e**) Activities of placental and plasma EST in GD12.5 and GD15.5 control mice and 100-TCS mice. **P < 0.01 *vs*. GD12.5 control mice; ^##^*P* < 0.01 *vs*. GD15.5 control mice (two way ANOVA). (**f**) Representative immunoblotting analysis showing the levels of plasma EST protein in control, low-TCS and high-TCS abortion patients. *P < 0.05 and **P < 0.01 *vs*. control group (one way ANOVA). The blots in panel band were performed on the same blot membranes and shown as cropped images. Full-length versions of all western blots are presented in Supplementary Figure 1. (**g**) Activity of plasma EST in control, low-TCS and high-TCS abortion patients. **P < 0.01 *vs*. control group; ^#^*P* < 0.05 *vs*. low-TCS abortion patients (one way ANOVA).

The level of placental *SULT1E1* mRNA in control mice was increased starting from GD10.5 and became highly abundant at GD15.5 (Fig. 3a). In comparison with control mice, the levels of placental *SULT1E1* mRNA in 100-TCS mice did not differ significantly at GD11.5 (*P* = 0.121, n = 10), GD12.5 (*P* = 0.236, n = 10) and GD13.5 (*P* = 0.063, n = 10) followed by a detectable decline at GD14.5-16.5 (*P* < 0.001, n = 10). Consistently, the levels of EST protein in placenta (Fig. 3b) or plasma (Fig. 3c) in GD15.5 100-TCS mice were lower than those in control mice (*P* < 0.001, n = 10), although had no significant difference between GD12.5 100-TCS mice and control mice (placenta: *P* = 0.162, n = 10; plasma: *P* = 0.085, n = 10). The activity of EST was further examined by radiochemical extraction assay when the amount of EST protein was corrected to be equal. Notably, the activities of placental and plasma EST in either GD12.5 (*P* < 0.001, n = 10; Fig. 3d,e) or GD15.5 100-TCS mice (*P* < 0.001, n = 10) were significantly reduced compared to control mice.

In comparison with control group of normal gestation 22–24 weeks, the level of plasma EST protein was reduced in low-TCS (*P* = 0.013, n = 10; Fig. 3f) and high-TCS abortion patients (*P* < 0.001, n = 10). Although the EST protein in high-TCS abortion patients tended to be less than that in low-TCS abortion patients, the group comparison failed to reach significance (*P* = 0.111, n = 10). Importantly, the activity of plasma EST in high-TCS abortion patients was lower than in control group (*P* < 0.001, n = 10; Fig. 3g) and low-TCS abortion patients (*P* = 0.027, n = 10).

### TCS-exposure enhances platelet aggregation to cause placental thrombosis

**Figure 4:**
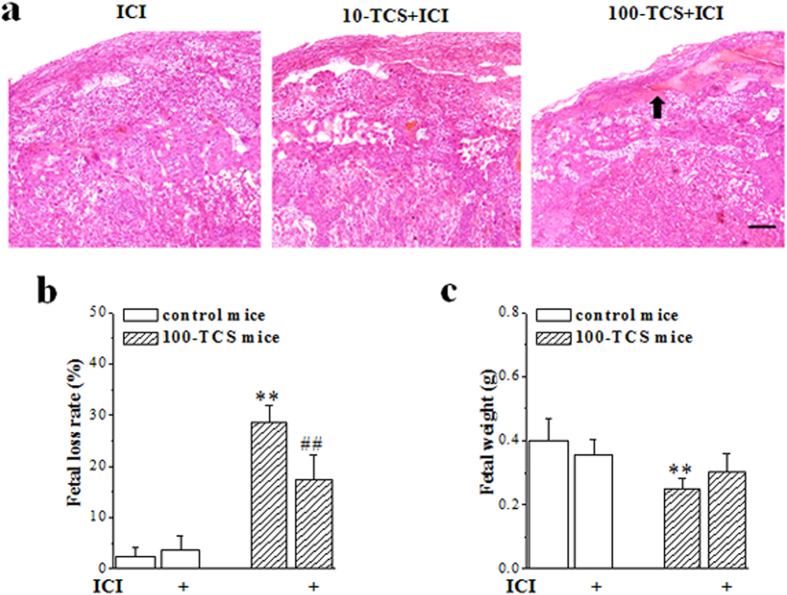
Effects of ER antagonist in TCS-induced placental thrombosis and spontaneous abortion. (**a**) Effects of ICI 182,780 (ICI) on TCS-caused placental thrombosis in GD15.5 TCS mice. Block arrow indicates thrombi. Scale bars = 100 μm. (**b,c**) Effects of ICI 182,780 (ICI) on fetal loss and reduced body weight (g) of live fetuses in GD15.5 100-TCS mice. **P < 0.01 *vs*. control mice; ^##^*P* < 0.01 *vs*. 100-TCS mice (two way ANOVA).

To test whether the TCS-induced increase in activated E2 is involved in the placental thrombosis and spontaneous abortion, we examined blood coagulation state in GD15.5 100-TCS mice. The results showed that the levels of plasma fibrin degradation product (FDP) (*P* = 0.002, n = 8; Table 4) and ADP-induced platelet aggregation (*P* = 0.020, n = 8) were increased in 100-TCS mice with the decline of prothrombin time (PT) (*P* = 0.019, n = 8) and activated partial thromboplastin time (APTT) (*P* = 0.032, n = 8) compared to controls, which could be corrected by the estrogen receptor (ER) antagonist ICI 182,780 when administered on GD12.5-15.5 (FDP: *P* = 0.009, n = 8; ADP-induced platelet aggregation: *P* = 0.042, n = 8; PT: *P* = 0.005, n = 8; APTT: *P* = 0.048, n = 8). As expected, the administration of ICI 182,780 prevented the placental thrombosis in GD15.5 10-TCS and 100-TCS mice (Fig. 4a) and partially reduced the fetal death in 100-TCS mice (*P* < 0.001, n = 10; Fig. 4b). Although the blockade of ER by ICI 182,780 had a tendency to rescue the decline of fetal body weight, the group comparison with vehicle-treated 100-TCS mice failed to reach significance (*P* = 0.066, n = 10; Fig. 4c).

## Discussion

The present study provides the first *in vivo* evidence that exposure to TCS can cause spontaneous abortion in humans and mice. This conclusion is deduced mainly from the following results. The level of urinary TCS in 28.3% high-TCS spontaneous abortion patients (11.21 ng/ml) was increased by 11.3-fold compared to normal pregnancies (0.99 ng/ml). The National Health and Nutrition Examination Survey collected 2,517 urine samples in a representative US population and detected urinary TCS in 74.6% of the samples at concentrations of 2.4–3790 μg/l^7^. Based on species differences, by conversion of an uncertainty factor (100 times), we inferred that the level of urinary TCS in mice exposed to 10 mg/kg TCS was equivalent to that in high-TCS abortion patients, while in mice exposed to 100 mg/kg TCS was equivalent to the high exposure level of US population. According to the level of urinary TCS in abortion patients, we prepared the model of mice exposed to TCS from gestation day (GD) 5.5. More importantly, the exposure to TCS at doses of 10 and 100 mg/kg in pregnancy mice caused an approximately 60–80% spontaneous abortion with the growth retardation of fetuses during GD12.5–16.5.

*SULT1E1* expression has been determined in the placenta of humans, cows and mice during mid- to late gestation^13^,^14^,^21^. Consistent with an earlier report by Tong *et al.*^16^, the expression of placental EST in mice was increased from GD12.5 and reached an approximately 6-fold increase at GD15.5. In 100-TCS mice, the levels of placental *SULT1E1* mRNA and EST protein in GD12.5 did not differ significantly from control mice, while in GD15.5 were reduced compared to controls. One possibility is that the placental tissue necrosis in GD15.5 100-TCS mice leads to the reduction of EST. In addition, the level of plasma EST protein had no significant difference between low-TCS and high-TCS abortion patients, although it was lower than that in control group. It is conceivable that the TCS-exposure does not affect the EST expression in mice and human. One principal finding in this study is that the activity of EST was significantly reduced in high-TCS abortion patients and GD12.5-15.5 TCS mice. James *et al.* have reported that the inhibitory effect of TCS on sheep placental EST activity was mixed competitive/uncompetitive actions^12^. Moreover, a potent human EST inhibitor 4′OHCB79^11^ is a highly potent inhibitor of sheep EST^12^, providing evidence that animal and human EST respond similarly to TCS inhibition. Therefore, our results suggest that the exposure of TCS can inhibit the activity of EST in humans and mice.

Placenta provides nearly all of E2 in the forms of sulfo-conjugated E2 (S-E2) and estrone, which are present at much higher concentrations than the unconjugated E2 (free E2). The discrete localization of EST at the interface of the fetal-maternal blood exchange suggests that EST may play a critical role in modulating E2 activity in both fetal and maternal plasma. The levels of serum E2 in *SULT1E1* knockout mice from DG12.5 to DG 17.5 were significantly elevated, which were associated with the increase in the levels of free E2 in the amniotic fluids at DG14.5 and DG17.5^17^. Thus, it is conceivable that the decline of EST activity during mid- to late gestation through reducing the degradation and excretion of E2 can elevate the level of activated E2. Although the levels of serum E2 at DG12.5 and DG15.5 had no difference between TCS mice and control mice, the ratio of S-E2 and free E2 in TCS mice was lower than that in control mice, implying an increase of the activated E2 in TCS mice. It is possibility that the decline of serum P4 level in TCS mice arises from the placental degeneration and failure.

Another important finding in the present study is that the platelet aggregation and platelet activation were enhanced in TCS mice, which could be blocked by the ER antagonist. In particular, the blockade of ER reduced remarkably the placental thrombus and hemorrhage in GD15.5 TCS mice. The over-activation of ER has been reported to induce platelet aggregation in human through cascading Src, Pyk2 and PI3-kinase or increasing the integrin affinity for fibrinogen to reduce the threshold of platelet activation^22^. E2 intake in women can increase the procoagulant factors and decrease the anticoagulant factors^23^,^24^,^25^. In addition, the *SULT1E1* knockout mice showed placental thrombus and fetal death during mid- to late gestation^16^,^21^, in which the treatment with E2 is able to exacerbate the fetal loss phenotype in a dose-dependent manner. Furthermore, higher dose of E2 has been demonstrated to cause placental thrombosis and an increase in fetal death incidence in wild-type mice. Therefore, our results give an indication that the inhibition of EST by TCS enhances the platelet aggregation and platelet activation through increasing activated E2 to create a procoagulant state and cause the placental hemorrhage and necrosis.

Interestingly, the ER antagonist could attenuate the fetal death in TCS mice. A possible explanation is that the increase of activated E2 in TCS mice causes the placental degeneration and failure leading to spontaneous abortion. The delivery of oxygen and nutrients to the developing fetus is highly dependent on the maintenance of high uterine blood flow. Wood *et al.* have reported the presence of estradiol-3-sulfate in the plasma of fetal sheep at concentrations of approximately 1 ng/ml^16^. The sulfated estrogen formed in the placenta is transferred to the fetus, as a major source of E2^26^. In E2-responsive tissues, steroid sulfatase can hydrolyze sulfo-conjugated E2 into active E2^27^. It is clear that E2 is required for normal fetal development^28^,^29^. E2 potently stimulates fetal ACTH secretion^30^. The increase in fetal ACTH and cortisol concentrations can further elevate fetal stress responsiveness and accelerate fetal maturation. In addition, E2 could stimulate thyroid growth by elevating the expression of thyrotropin (TSH)^31^. Therefore, it is proposed that TCS through suppressed EST activity leads to the decline in the transformation of circulating E2 from maternal plasma to the fetus, delaying fetal development and maturation. On the other hand, the exposure to TCS (30 mg/kg) only can decrease the level of serum T4 in male rats^8^. An earlier study^9^ reported that the exposure to TCS (10–50 mg/kg) could decrease the serum total T4 and T3 in pregnant rats, which contributes to the diminished growth and development of fetuses^32^. Our results showed the decline of serum T3 and T4 in TCS mice. Because the ER antagonist partially rescued the decline of fetal body weight in TCS mice, thus it is indicated that the reduction of thyroid hormone in TCS mice is an initiating event for the growth retardation of fetuses.

TCS is widely used in personal care products. Stanley *et al.* reported that the concentration of TCS that causes substantial inhibition of EST is in the range of the environmental exposure level^14^. The present study provides *in vivo* evidence that the exposure of human or mice to TCS, at least partially, through inhibiting EST activity might cause spontaneous abortion and growth retardation of fetuses. This study is the first to implicate that the activation of EST is required for the maintenance of pregnancy and fetal development during mid to late gestation in human.

## Materials and Methods

### Collection of spontaneous abortion cases

The subjects were volunteers from the NJMU Birth Cohort at affiliated hospitals of Nanjing Medical University. The Institutional Review Board of Nanjing Medical University approved the protocols. All of the studies involving human subjects were performed under full compliance with government policies and the Helsinki Declaration. After explanation of the study procedures and clarification of the questions raised, all of the subjects gave their informed consent. A complete physical examination, including measurement of the height and weight, was performed. A questionnaire was used to collect information, including personal background, lifestyle factors, occupational and environmental exposures, genetic risk factors, sexual and reproduction statuses, medical history, and physical activity. Controls were females who had no history of spontaneous abortion and had at least one living child. The cases were females who had medically unexplained spontaneous abortion in mid-gestation weeks 14–24^33^. We excluded the subjects with certain known factors related to spontaneous abortion, such as chromosomal abnormality, uterine abnormalities, autoimmune diseases, infection, and occupational exposure to certain toxins suspected to be associated with spontaneous abortion from this study. All of the participants for final analyses, including 113 eligible cases and 339 eligible controls in mid-gestation weeks 14–24, claimed that their life styles and environment had not changed for several months leading up to sample collection. Urine, serum and plasma samples were collected within 12 hours after spontaneous abortion. These samples were frozen at −20 °C until phenol analysis.

### Measurement of urinary TCS

The urinary concentration of TCS was measured with ultra-high performance liquid chromatography-tandem mass spectrometry (UPLC-MS/MS) (Milford, MA, USA). Firstly, urine samples were incubated in 1 M ammonium acetate buffer solution (pH = 5.0) for hydrolyzation with β-glucuronidase/sulfatase (20000 units/ml) overnight. After hydrolysis, the phenols were extracted and preconcentrated with solid phase extraction (SPE) (500 mg/3 ml, Supelclean, USA), and determined with UPLC (Acquity UPLCTM BEH C18 column, 1.7 μm, 2.1 × 100 mm) electrospray ionization (negative ion mode)-MS/MS. The limit of detection (LOD) was 0.9 ng/ml. The intra- and inter-day precisions for TCS were between 9% and 38% and the recoveries were between 99% and 138% at spiked concentration of 2, 20 ng/mL. Quality control samples were analyzed in parallel with unknown samples in each analytical series. Creatinine (CR) concentrations were analyzed for correcting the phenols concentration variations caused by fluctuated urine concentration and dilution. CR concentrations of urine were measured with an automated chemistry analyzer (7020 Hitachi, Japan).

### Animals and drug administration

All of the animal experimental procedures followed the guidelines of the Laboratory Animal Research of Nanjing Medical University and were approved by the Institutional Animal Care and Use Committee of Nanjing Medical University. Three-month-old female and male mice (Oriental Bio Service Inc., Nanjing), weighing approximately 30–35 g, were used in the study. All of the mice were housed under a 12/12 hour light/dark cycle (lights on at 0600 hour) with free access to food and tap water. Detection of a vaginal plug was chosen to indicate day 0.5 of gestation (GD0.5). TCS (Sigma, St. Louis, Mo.) was dissolved in dimethylsulfoxide (DMSO), and then diluted was with corn oil. The dams were given oral administration of TCS at doses of 1, 10 and 100 mg/kg per day. Control mice were treated with vehicle at the same volume. During the treatment with TCS, the dams did not show significant abnormality in body weight. A pellet of the ER antagonist ICI182,780 (Sigma, St. Louis, Mo.) with 1.5 mg/12 days of release^16^ was implanted at GD12.5. Control mice received placebo pellet implantation.

### Histological examination

The placenta was fixed in 4% paraformaldehyde and then dehydrated through a graded series of alcohol, cleared in xylene and embedded in paraffin wax. Sections (5-μm thick) deparaffinized were stained with hematoxylin and eosin (HE) and observed using a conventional light microscope (Olympus DP70; Tokyo, Japan).

### Measurement of serum hormones

The blood samples were collected within 12 hours after spontaneous abortion in humans. Orbital blood (500 μl) of mice was obtained under anesthetized conditions with chloralhydrate (400 mg/kg, i.p.) at 9 hours (1600–1700) after the administration of TCS. Levels of serum total E2, progesterone (P4), β-human chorionic gonadotropin (β-HCG), thyroxine (T4) and triiodothyronine (T3) were examined using a radioimmunoassay (RIA) kit provided by the National Hormone and Peptide Programme (Baltimore, MD). The intra- and inter-assay coefficients of variation were 6% and 5.8% for E2, 5.8% and 8.4% for P4, 3.4% and 12.3% for β-HCG, 5.1% and 8.6% for T4, 2.9% and 8.3% for T3, respectively. The lowest detectable levels were 2 pg/ml for E2, 0.2 ng/ml for P4, 2 mIU/ml for β-HCG, 5.0 ng/ml for T4, 0.1 ng/ml for T3, respectively. The plasma was extracted with ethanol to preserve the water-soluble steroid including sulfo-conjugated E2 (S-E2) or using hexane/ethyl acetate (3:2, vol/vol) to obtain unconjugated E2 (free E2), according to the method of Wood *et al.*^16^. Then, the levels of S-E2 and free E2 in extraction were measured by enzyme-linked immunoassay (Oxford Chemical Co., Oxford, MI). The intraassay coefficient of variation in this enzyme-linked immunoassay was 8.1%.

### Assessment of coagulation state

The anticoagulated blood samples using sodium citrate were collected. Fibrin degradation product (FDP) is measured by Cusabio rat FDP ELISA assay kit (Cusabio, Wuhan, China) according to the manufacturer’s instructions. To estimate platelet aggregation, platelet rich plasma (PRP) was prepared by centrifugation (250 g) for 10 min, and then tested by the turbidimetric technique according to the method of Mustard *et al.*^33^. The platelet aggregation was induced using ADP at a final concentration of 10 μM. The prothrombin time (PT) and activated partial thromboplastin time (APTT) were measured using calcium rabbit brain thromboplastin and kaolin platelet substitute techniques (Diagen Diagnostic Reagent Ltd., Oxon, UK) 33.

### Western blot analysis

We performed Western blot analysis as described previously^34^. Briefly, the placenta was homogenized to obtain protein samples. Next, proteins (50 μg) were resolved by SDS-PAGE, transferred onto PVDF membrane, probed with a rabbit anti-mouse EST polyclonal antibody (1:200; Santa Cruz Biotechnology, CA, USA), and labeled with an HRP-labeled goat anti-rabbit IgG (1:5000; Millipore, Billerica, MA). Signal quantification was carried out using Quantity One image software (Bio-Rad).

### Reverse transcription quantitative polymerase chain reaction (RT-qPCR)

Total RNA was extracted from the placentas using Trizol (Invitrogen, Carlsbad, CA, USA) according to the manufacturer’s instructions. RNA (1 μg) was used for reverse transcription using high-capacity cDNA of the reverse transcription kit RT (TaKaRa Biotechnology CO., Ltd) according to the instructions. The primer sequences of *SULT1E1* and *GAPDH* mRNA were designed according to earlier publications^34^. RT-qPCR was performed using a Light Cycler Fast Start DNA Master SYBR Green I kit and an ABI Prism 7300 Sequence Detection System (Applied Biosystems, Foster City, California, USA), and the relative expression of genes was determined using the 2-ΔΔct method with normalization to GAPDH expression. The results were averaged from four sets of independent experiments.

### Assays of EST activity

EST activities were measured using a radiochemical extraction assay^35^. 3′-Phosphoadenosine-5′-phosphosulfate was purchased from Sigma-Aldrich chemical company (St. Louis, MO), and ^3^H-estradiol (60 Ci/mmol) was purchased from PerkinElmer Life Sciences Inc. (Boston, MA). Incubation mixtures (0.5 ml) contained 100 mM Tris–HCl (pH 7.4), 5 mM MgCl_2_, 0.8 mg placental cytosolic protein, 20 μM PAPS, and 2 nM [^3^H] E2. The reaction was initiated with the addition of PAPS and continued for 30 minutes at 37 °C. The reaction mixture was extracted with 1 ml of dichloromethane and aliquot of the aqueous phase was counted by liquid scintillation counting.

### Statistical analysis

The group data were expressed as the means ± standard error (SE). All of the statistical analyses were performed using the SPSS 16.0 software (SPSS Inc., Chicago, IL, USA). Differences among the means were analyzed using ANOVA, followed by Bonferroni *post-hoc* analysis, or Student’s *t* test when appropriate. In some cases, logistic regression and chi-squared test were used. Differences at *P* < 0.05 were considered statistically significant.

## Additional Information

**How to cite this article**: Wang, X. *et al.* Triclosan causes spontaneous abortion accompanied by decline of estrogen sulfotransferase activity in humans and mice. *Sci. Rep.* **5**, 18252; doi: 10.1038/srep18252 (2015).

## Supplementary Material

Supplementary Information

## Figures and Tables

**Table 1:** Distribution of unadjusted TCS level (ng/ml) in urine.

**chemical TCS**	**detection rate (%)**	**geometric mean (95% CI)**	**selected percentiles**			
			**50**^**th**^	**75**^**th**^	**90**^**th**^	**95**^**th**^
abortion	57.52	2.50 (1.49–3.51)	1.40	5.43	37.39	95.94
control	32.74	0.99 (0.86–1.15)	<LOD	1.65	8.28	23.56

The LOD of TCS was 0.9 ng/ml. All values below LOD were assigned a value equal to the LOD divided by 2. CI: confidence interval.

**Table 2:** Crude and adjusted ORs (95% CIs) for spontaneous abortion by the exposure level of TCS.

**chemical TCS**	**control No.**	**abortion No.**	**crude**		**adjusted**	
			**OR(95%CI)**	**P-value**	**OR(95%CI)**^a^	**P-value**
low	228	48	1.00		1.00	
median	55	33	2.85(1.67-4.85)*	1.13E-04	2.00(1.08-3.70)*	0.027
high	56	32	2.71(1.59-4.63)*	2.52E-04	2.36(1.29-4.34)*	0.006
P-value for trend				2.34E-05		0.003

^a^ORs are adjusted for BMI, drinking status and creatinine level. *P < 0.05 vs. low-TCS exposure level.

**Table 3:** Levels of hormones in serum.

**hormones**	**human**			**mice**			
	**control**	**abortion**		**GD12.5**		**GD15.5**	
		**low-TCS**	**high-TCS**	**control**	**100-TCS**	**control**	**100-TCS**
E2(pg/ml)	238.77	165.69*	201.18	15.52	18.42	24.81##	20.70
free E2 (pg/ml)	–	–	–	3.18	5.57#	5.23#	6.85+
S-E2 (pg/ml)	–	–	–	17.24	8.60##	25.88	9.92++
P4 (pg/ml)	75.84	27.24**	20.88**	11.90	10.13	21.13##	6.17++
β-HCG (mIU/ml)	35.26	11.07**	8.75**	5.73	4.72	7.09	2.59++
T4 (ng/ml)	–	–	–	21.08	12.83#	19.51	9.13^+^
T3 (ng/ml)	–	–	–	1.76	0.88#	2.10	1.17+

*P < 0.05, **P < 0.01 *vs*. control. ^#^*P* < 0.05, ^##^*P* < 0.01 *vs*. GD12.5 control. ^+^*P* < 0.05,^++^*P* < 0.01 *vs*. GD15.5 control.

**Table 4:** Coagulation state in GD15.5 mice.

	**control**	**100-TCS**	**ICI**	**100-TCS/+ICI**
FDP (ng/ml)	15.25 ± 2.50	24.67 ± 1.53**	11.91 ± 2.83	17.50 ± 0.97##
PT (s)	17.60 ± 0.88	14.23 ± 1.74*	21.38 ± 2.19*	19.80 ± 1.56##
APPT (s)	22.25 ± 2.38	16.40 ± 2.88*	25.31 ± 3.44	23.55 ± 3.03#
platelet aggregation (%)	32.75 ± 3.40	59.33 ± 9.06*	27.51 ± 2.51	34.50 ± 5.39#

*P < 0.05, **P < 0.01 *vs*. control. ^#^*P* < 0.05, ^##^*P* < 0.01 *vs*. 100-TCS mice.
