# Supplementary material for: Triclosan causes spontaneous abortion accompanied by decline of estrogen sulfotransferase activity in humans and mice
Source: Sci Rep. 2015 Dec 15;5:18252. doi: 10.1038/srep18252 (PMC4678904; doi:10.1038/srep18252)
Supplement: Supplementary Information [file srep18252-s1.pdf]

Supplementary Information

Triclosan causes spontaneous abortion accompanied by decline of strogen sulfotransferase activity in humans and mice

Short title: TCS-caused spontaneous abortion

#Xiaoli Wang[1,2], #Xiaojiao Chen[1,3,4], Xuejiao Feng[2], Fei Chang[2], Minjian Chen[1,4], Yankai Xia[1,3,4]*, Ling Chen[1,2]*

[1]State Key Lab of Reproductive Medicine, [2]Department of Physiology, [3]Nanjing Maternity and Child Health Hospital, [4]Key Laboratory of Modern Toxicology of Ministry of Education, School of Public Health, Nanjing Medical University, Nanjing 210029, China,

*Corresponding author: Ling Chen, Ph.D. & M.D.

Address: Department of Physiology, Nanjing Medical University, Hanzhong Road 140, Nanjing, China.

Tel: +86-25-86862878, Fax: +86-25-86260332

E-mail: lingchen@njmu.edu.cn

*Corresponding author: Yankai Xia, Ph.D.

Address: State Key Laboratory of Reproductive Medicine, Institute of Toxicology, Nanjing Medical University, 818 East Tianyuan Road, Nanjing 211166, China.

Tel: +86-25-86868425, Fax: +86-25-86868427

E-mail: yankaixia@njmu.edu.cn

Supplementary Figure 1

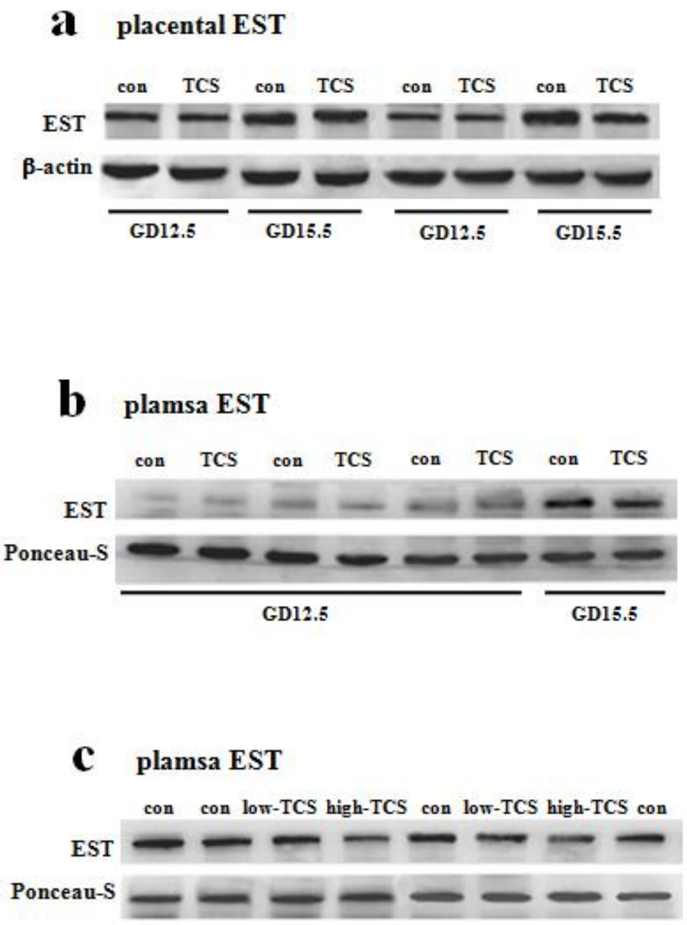

Supplementary Figure 1 (**a**) Full-length blots/gels of placental EST protein in GD12.5 and GD15.5 control mice and 100-TCS mice in the Figure 3b in the main paper. (**b**) Full-length blots/gels of plasma EST protein in GD12.5 and GD15.5 control mice and 100-TCS mice in the Figure 3c in the main paper. (**c**) Full-length blots/gels of plasma EST protein in control, low-TCS and high-TCS abortion patients in the Figure 3f in the main paper.
